# Update of IGF-1 receptor inhibitor (ganitumab, dalotuzumab, cixutumumab, teprotumumab and figitumumab) effects on cancer therapy

**DOI:** 10.18632/oncotarget.15704

**Published:** 2017-02-25

**Authors:** Xiao Qu, Zhinan Wu, Wei Dong, Tiehong Zhang, Liguang Wang, Zhaofei Pang, Wei Ma, Jiajun Du

**Affiliations:** ^1^ Department of Oncology, Shandong Provincial Hospital Affiliated to Shandong University, Shandong, China; ^2^ Institute of Oncology, Shandong Provincial Hospital Affiliated to Shandong University, Shandong, China; ^3^ Department of Thoracic Surgery, Shandong Provincial Hospital Affiliated to Shandong University, Shandong, China

**Keywords:** IGF-1R, combination chemotherapy, prognostic clinical trials, cancer treatment, curative effects

## Abstract

**Background:**

Prognostic studies of insulin-like growth factor-1 receptor(IGF-1R) inhibitors in cancer therapy had promising results in infratests, which exhibited that IGF-1R signalling was crucial in cancer cells growth. However, the conclusion of later clinical trials revealed a dim future for IGF-1R inhibitors to treat cancer. We conducted this analysis to figure out how IGF-1R inhibitors acted in clinical cancer therapy.

**Material and Methods:**

We searched up-to-date studies about the single agent of IGF-1R inhibitors or combination with other therapies in solid tumor. Five IGF-1R anti-agents were involved. The primary endpoint was progression-free survival (PFS). The secondary endpoint was overall survival (OS).

**Result:**

17studies were enrolled. The results was not significant in overall survival (I^2^=37.1%, P=0.080, HR=1.08, 95% CI=0.97-1.21) and in progression-free survival (I^2^=0.0%, P=0.637, HR=1.05, 95% CI=0.98-1.12). OS for dalotuzumab, breast cancer, colorectal cancer, and PFS for prostate cancer even indicated harmful effects.

**Conclusion:**

So far, anti-IGF-1R mono-antibodies did not make significant differences in solid tumor prognosis. On the contrary, pessimistic effects were shown in the dalotuzumab, breast cancer, colorectal cancer and prostate cancer subgroups. Further studies of IGF-1R anti-agents were needed, but unwarranted in unselected patients by predictive biomarkers.

## INTRODUCTION

Cancers are series of diseases possessing high mortality in America, in which lung cancer, prostate cancer, breast cancer, colorectal cancer, ovarian cancer, and pancreatic cancer are mostly rangking forward [1]. Insulin-like growth factor-1 receptor (IGF-1R) induces the common pathways for normal cell growth, as well as cancer development, suggesting that IGF-1R is a potential target for cancer therapy [2, 3]. Various strategies have been used to target components of IGF-1R system, including small interfering RNA, antisense oligonucleotides, antisense RNA, triple helix-forming oligodeoxynucleotides, specific kinase inhibitors, single chain antibodies and fully humanized anti-IGF1R monoclonal antibodies [4]. Two of the most prevalent strategies are small-molecule tyrosine kinase inhibitors and monoclonal antibodies [5, 6]. Ganitumab (AMG-479), dalotuzumab (MK-0646), cixutumumab (IMC-A12), teprotumumab (R1507), and figitumumab (CP-751,871) are commonly used recombinant, fully human monoclonal antibodies against the insulin-like growth factor 1 receptor (IGF-1R). [7] These agents prevent binding of IGF-1 to IGF1R and subsequently inhibit down stream signaling, including PI3K/Akt pathway. [8, 9] PI3K-Akt Pathway can promote cell survival and growth in response to extracellular signals. It is highly regulated by multiple mechanisms, often involved in cross-talk with other signal pathways. [10] Therefore, inhibition of IGF-1R signaling and subsequent pathway may result in the inhibition of tumor cell proliferation and the induction of tumor cell apoptosis. [8, 11]

Up to date, outcomes of clinical studies about IGF-1R inhibitors seems to be unsatisfactory. We found only one study [12] seemed to have the active trend that IGF-1R inhibitors (AMG-479) improved the PFS or OS in advanced solid tumors. Some studies [13–15] revealed IGF-1R inhibitors could shorten OS and PFS. However, more studies [16–25] showed IGF-1R mono-antibodies had no significant value in cancer treatment. Three data from ongoing clinical trials (NCT00372996, 2015; NCT00887159, 2015; NCT00684983, 2016) also indicated insignificant cancer curative value of anti-IGF-1R agents. Herein, we conducted this meta-analysis by merging some similar study data. And overall and subgroup outcomes elucidated the situation of curative effects of these five anti-IGF-1R agents for patients with solid tumors. It should be noted that this analysis was designed to estimate the effect of the treatment with the combination of IGF-1R anti-agents and standard chemotherapy protocol. Thus statistically insignificant result was regarded as meaningful outcome as well. This meta-analysis was performed with up-to-date data.

## RESULTS

### Inclusion procedure

**Figure 1:**
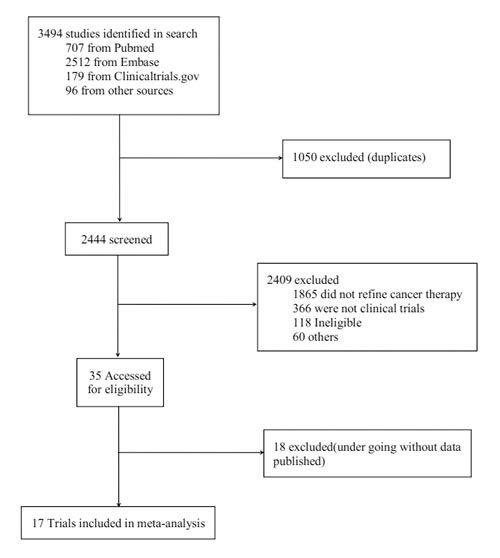
The Flow Chart of Study Selection

A total of 17 studies were enrolled to evaluate the curative effects of IGF-1R inhibitors for patients with solid tumors. These studies [12–14, 16–22] (NCT00372996, 2015; NCT00887159, 2015; NCT00684983, 2016) were selected according to the process shown in Figure 1. 3494 studies were identified in search, in which 707 were from Pubmed, 2512 from Embase, 179 from Clinicaltrials.gov, and 96 from other sources. The elementary screening excluded 1050 duplicates and 2444 studies were left to the second screening. After the second screening, 35 studies were accessed for eligibility. Further selection excluded 18 studies that were undergoing without data published. Finally, 17 studies were enrolled into analysis.

### Risk of bias assessment

Our assessment result of risk of bias was shown in Table 1. Most included studies were assessed as unclear risk of bias. One study [14] was assessed as low risk. Two studies [12, 24] were high risk.

**Table 1:** Summary of bias of included studies

Study ID	study	Random sequence generation	Allocation concealment	Blinding of participants and personnel	Blinding of outcome assessment	Incomplete outcome data	Selective reporting	Anything else, ideally prespecified	Overall bias
01	John F R Robertson et al., 2013	low	low	low	low	low	low	low	low
02	Francesco Sclafani et al., 2015	unclear	unclear	unclear	unclear	low	low	unclear	unclear
03	H. L. Kindler et al., 2012	low	high	low	unclear	low	low	unclear	high
04	C. S. Fuchs et al., 2015	unclear	unclear	unclear	unclear	low	low	unclear	unclear
05	G. V. Scagliotti et al., 2014	low	unclear	unclear	unclear	low	unclear	unclear	unclear
06	Suresh S. Ramalingam et al., 2011	unclear	unclear	unclear	unclear	low	unclear	unclear	unclear
07	Philip A. Philip et al., 2015	low	unclear	low	unclear	low	unclear	unclear	unclear
08	Teresa Moran et al., 2014	unclear	unclear	unclear	unclear	unclear	unclear	unclear	unclear
09	Corey J. Langer et al., 2014	unclear	unclear	unclear	unclear	unclear	unclear	unclear	unclear
10	Nasser H. Hanna et al., 2015	unclear	unclear	high	high	low	unclear	unclear	high
11	Johann S. de Bono et al., 2014	unclear	unclear	unclear	unclear	unclear	unclear	unclear	unclear
12	A. L. Cohn et al., 2013	unclear	unclear	unclear	unclear	unclear	unclear	unclear	unclear
13	Eric Van Cutsem et al., 2015	low	low	low	low	unclear	unclear	unclear	unclear
14	Gottfried E. Konecny et al., 2014	unclear	unclear	unclear	unclear	unclear	unclear	unclear	unclear
15	NCT00372996, 2015	unclear	unclear	unclear	unclear	unclear	unclear	unclear	unclear
16	NCT00887159, 2015	unclear	unclear	unclear	unclear	unclear	unclear	unclear	unclear
17	NCT00684983, 2016	unclear	unclear	unclear	unclear	unclear	unclear	unclear	unclear

Low: Low risk of bias; Unclear: Unclear risk of bias; High: High risk of bias. The assessment was based on the Cochrane Collaboration's tool. John F R Robertson et al., 2013 was assessed the best quality. The rest studies were assessed as unclear with exception of two high-risk studies(Nasser H. Hanna et al., 2015; H. L. Kindler et al., 2012).

### Main characteristics of included studies

**Figure 2:**
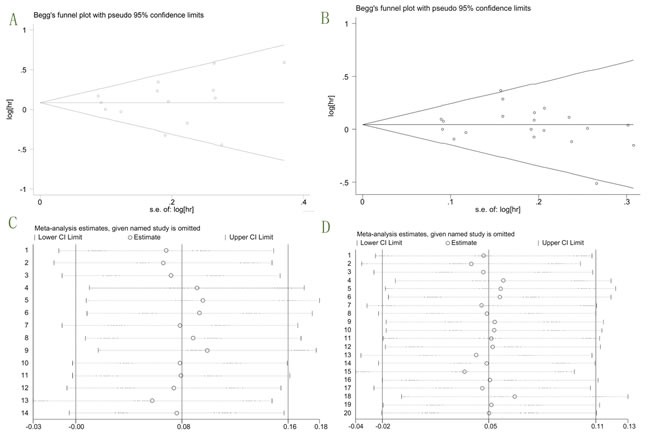
Begg funnel plots and sensitivity analysis **A**. Begg funnel plot for overall OS; **B**. Begg funnel plot for overall PFS; **C**. Sensitivity analysis for OS; **D**. Sensitivity analysis for PFS.

The basic characteristics of the 17 enrolled studies were listed in Table 2. Of the enrolled studies, there were three of them with data exhibited in Clinicaltrials.gov but without formal article published (NCT00372996, 2015; NCT00887159, 2015; NCT00684983, 2016), while the other 14 with full articles. 3 studies contained two sets of data [15, 16, 23]. Three datas from two studies presented their confidental interval (CI) in proportion of 80% and 90%. We used stata to calculate their 95% CI as well as hazard ratio (HR). The published Year of enrolled studies ranged from 2012 to 2016. Sample size varied from 64 to 800. There were six types of cancer included: breast cancer, colorectal cancer, pancreatic cancer, lung cancer, prostate cancer, and ovarian cancer. More details about these cancers are: advanced hormone-receptor-positive breast cancer [14] (NCT00372996, 2015), metastatic colorectal cancer [15], wild-type KRAS metastatic colorectal cancer [21], mutant KRAS metastatic colorectal cancer [22], metastatic pancreatic cancer [12, 20], metastatic adenocarcinoma of the pancreas [23], and advanced-stage non-small-cell lung cancer [16, 17, 19, 24, 25] (NCT00887159, 2015). Study phase information: 2 phase-Ib/II studies, 1 phase-I/II study, 7 phase-II studies, 3 phase-III studies, 1 phase-II/III study, and 3 unknown phase studies. We analysed the potential cause of heterogeneity by sensitivity analysis and Begg test (Figure 2). Symmetric funnel plot and t value of 0.28 and −0.38 for OS and PFS respectively indicated a low publication bias in both of them. The statistical results showed as (A) adj. Kendall's Score (P-Q) = 3; Std. Dev. of Score = 18.27; Number of Studies = 14; z = 0.16; Pr > |z| = 0.870; z = 0.11 (continuity corrected); Pr > |z| = 0.913 (continuity corrected); (B) adj. Kendall's Score (P-Q) = −36; Std. Dev. of Score = 30.82; Number of Studies = 20; z = −1.17; Pr > |z| = 0.243; z = 1.14 (continuity corrected); Pr > |z| = 0.256 (continuity corrected) respectively. The sensitivity analysis did not revealed any over resulted study. Therefore, in all, the publication bias of our analysis was basically accepted. During article searching, we found there were ongoing trials without data published (NCT01327612; NCT02306161; NCT01122199; NCT01061788; NCT01708161; NCT00791154; NCT01042379; NCT00769483; NCT01868997; NCT01232452; NCT00955305; NCT01142388). We recommend that the results of these trials should be followed up in time, so that more precise conclusion could be updated.

**Table 2:** Main characteristics of included studies

Study ID	study	Study phase	Pathologic Type	Patient number	NCT
01	John F R Robertson et al., 2013	II	Breast cancer	156	NCT00626106
02	Francesco Sclafani et al., 2015	II/III	Colorectal cancer	351	NCT00614393
03	H. L. Kindler et al., 2012	II	Pancreatic cancer	125	NCT00630552
04	C. S. Fuchs et al., 2015	III	Pancreatic cancer	800	NCT01231347
05	G. V. Scagliotti et al., 2014	III	Lung cancer	583	NCT00673049
06	Suresh S. Ramalingam et al., 2011	II	Lung cancer	171	NCT00760929
07	Philip A. Philip et al., 2015	Ib/II	Pancreatic cancer	200	NCT00617708
08	Teresa Moran et al., 2014	I/II	Lung cancer	75	NCT00654420
09	Corey J. Langer et al., 2014	III	Lung cancer	671	NCT00596830
10	Nasser H. Hanna et al., 2015	II	Lung cancer	90	NCT00986674
11	Johann S. de Bono et al., 2014	II	Prostate cancer	204	NCT00313781
12	A. L. Cohn et al., 2013	II	Colorectal cancer	104	NCT00813605
13	Eric Van Cutsem et al., 2015	Ib/II	Colorectal cancer	94	NCT00788957
14	Gottfried E. Konecny et al., 2014	II	Ovarian cancer	170	NCT00718523
15	NCT00372996, 2015	LA*	Breast cancer	219	NCT00372996
16	NCT00887159, 2015	LA*	Lung cancer	152	NCT00887159
17	NCT00684983, 2016	LA*	Breast cancer	64	NCT00684983

The situation of group allocation and patient demographics of studies were shown in Table 3. The 3 studies that contained two sets of data [15, 16, 23] had three pallel arms. And although the study Kindler HL et al.,2012 [12] also comprised three groups, the one with protocol “Conatumumab 10 mg/kg +gemcitabine” was not suitable for the second inclusion criteria. Of all the studies, the median age ranged from 53.0 to 68.9 years. The percentage of male was ranged from 32.0% to 78.0%, with exception of the studies that specifically enrolled patients by sexual index [14, 17, 18] (NCT00372996, 2015; NCT00684983, 2016). Three studies [14] (NCT00372996, 2015; NCT00684983, 2016) were conducted to discuss breast cancer, and one study [18] was to discuss ovarian cancer. Therefore, these four studies only adopted female patients. Study that only adopted male patients was de Bono JS et al.,2014 [17], which aimed at prostate cancer. Patients recieved exemestane, irinotecan, cetuximab, gemcitabine, erlotinib, panitumumab, paclitaxel+carboplatin (PC), cisplatin+etoposide (CE), lapatinib+capecitabine (LC), and docetaxel+prednisone (PD) as combination therapeutic protocols.

**Table 3:** Group allocation and patient demographics of studies

Study(ID)	Group	Age, mean & range	Sex		Race/ethnicity, n (%)			
			Male n(%)	Female n(%)	white	black	asian	other
01	(n=106)G	61.0(54–70)	0(0)	106(100)	100(94)	2(2)	3(3)	1(1)
	(n=50)Plb	62.0(55–66)	0(0)	50(100)	47(94)	2(4)	0(0)	1(2)
02	(n=119)iri+cet+Dal(weekly)	LA*	86(72.3)	33 (27.7)	0(0)	0(0)	54(45.4)	62(52.1)+3(2.5)A
	(n=119)iri+cet+Dal(2-weekly)	LA*	75(63.0)	44 (37.0)	0(0)	0(0)	59(49.6)	55(46.2)+5(4.2)A
	(n=116)iri+cet+Plb	LA*	82(70.7)	34 (29.3)	0(0)	0(0)	49(42.2)	60(51.7)+7(6.1)A
03	(n=42)G+gem	66.0(37–82)	25(60)	17 (40)	35(83)+CaucasianB	3(7)+AfricanC	0(0)	4(10)
	(n=41)Con+gem	61.0(45–80)	24(59)	17 (41)	32(78)+CaucasianB	3(7)+AfricanC	1(2)	5(12)
	(n=42)Plb+gem	61.0(43–82)	26(62)	16 (38)	37(88)+CaucasianB	3(7)+AfricanC	0(0)	2(5)
04	(n=322)Plb+gem	63.0(36–83)	188(58)	134 (42)	253(79)	3(1)	34(11)+30(9)D	1(<1)
	(n=318)G(12)+gem	62.0(36–85)	159(50)	159 (50)	258(81)	4(1)	19(6)+35(11)D	2(1)
	(n=160)G(20)+gem	62.0(31–81)	85(53)	75 (47)	129(81)	0(0)	14(9)+16(10)D	1(1)
05	(n=293)F	62.0(33–85)	228(78)	65(22)	249(85)	7(2)	21(7)	16(5)
	(n=290)Control	62.0(29–87)	225(78)	65(22)	238(82)	7(2)	23(8)	22(8)
06	(n=57)Er+Plb	62.0	20(35)	37(65)	55(96)	1(2)	0(0)	1(2)
	(n=57)Er+R1507(weekly)	63.0	18(32)	39(68)	55(96)	1(2)	0(0)	1(2)
	(n=57)Er+R1507(3-weekly)	62.0	19(33)	38(67)	56(98)	1(2)	0(0)	0(0)
07	(n=100)Er+gem+cix	63.0	40(40)	60(60)	LA*	LA*	LA*	LA*
	(n=100)Er+gem	64.0	59(59)	41(41)	LA*	LA*	LA*	LA*
08	(n=38)Er	59.0(36-80)	28(73.7)	10 (26.3)	0(0)	0(0)	2(5.3)	36(94.7)
	(n=37)Er+Dal	62.0(45-77)	27(73)	10 (27)	0(0)	0(0)	0(0)	37(100)
09	(n=342)PC+F	62.0(30-90)	261(76)	81(24)	265(78)	9(3)	56(16)	12(4)
	(n=339)PC	62.0(36-83)	260(77)	79(23)	270(80)	4(1)	59(17)	6(2)
10	(n=39)PC+cet	60.0(42-89)	20(51)	19 (49)	36(92)	3(8)	0(0)	0(0)
	(n=47)PC+cet+cix	60.0(44-76)	25(53)	22 (47)	44(94)	2(4)	0(0)	0(0)
11	(n=102)F+PD	68.9	102(100)	0(0)	94(92)	4(4)	0(0)	4(4)
	(n=102)PD	67.9	102(100)	0(0)	97(95)	2(2)	0(0)	3(3)
12	(n=52)G+FOLFIRI	58.0(28–81)	24(46)	28 (54)	41(79)	3(6)	7(13)	1(2)
	(n=52)Plb+FOLFIRI	59.0(32–80)	23(44)	29 (56)	38(73)	4(8)	9(17)	1(2)
13	(n=46)Pan+G	62.0(33–81)	25(54)	21(46)	LA*	LA*	LA*	LA*
	(n=48)Pan+Plb	55.0(19–75)	28(58)	20(42)	LA*	LA*	LA*	LA*
14	(n=85)PC	58.0(18-77)	0(0)	85(100)	LA*	LA*	LA*	LA*
	(n=85)PC+G	58.0(18-77)	0(0)	85(100)	LA*	LA*	LA*	LA*
15	(n=115)F+exe	61.2	0(0)	115(100)	LA*	LA*	LA*	LA*
	(n=104)exe	62.7	0(0)	104(100)	LA*	LA*	LA*	LA*
16	(n=48)CE	61.0(38-77)	25(52)	23(48)	LA*	LA*	LA*	LA*
	(n=52)CE+vis	64.0(52-87)	26(50)	26(50)	LA*	LA*	LA*	LA*
	(n=52)CE+cix	64.0(45-83)	25(48)	27(52)	LA*	LA*	LA*	LA*
17	(n=19)LC	57.0(35-75)	0(0)	19(100)	LA*	LA*	LA*	LA*
	(n=45)LC+cix	53.0(29-78)	0(0)	45(100)	LA*	LA*	LA*	LA*

### Overall outcomes of OS and PFS

**Figure 3:**
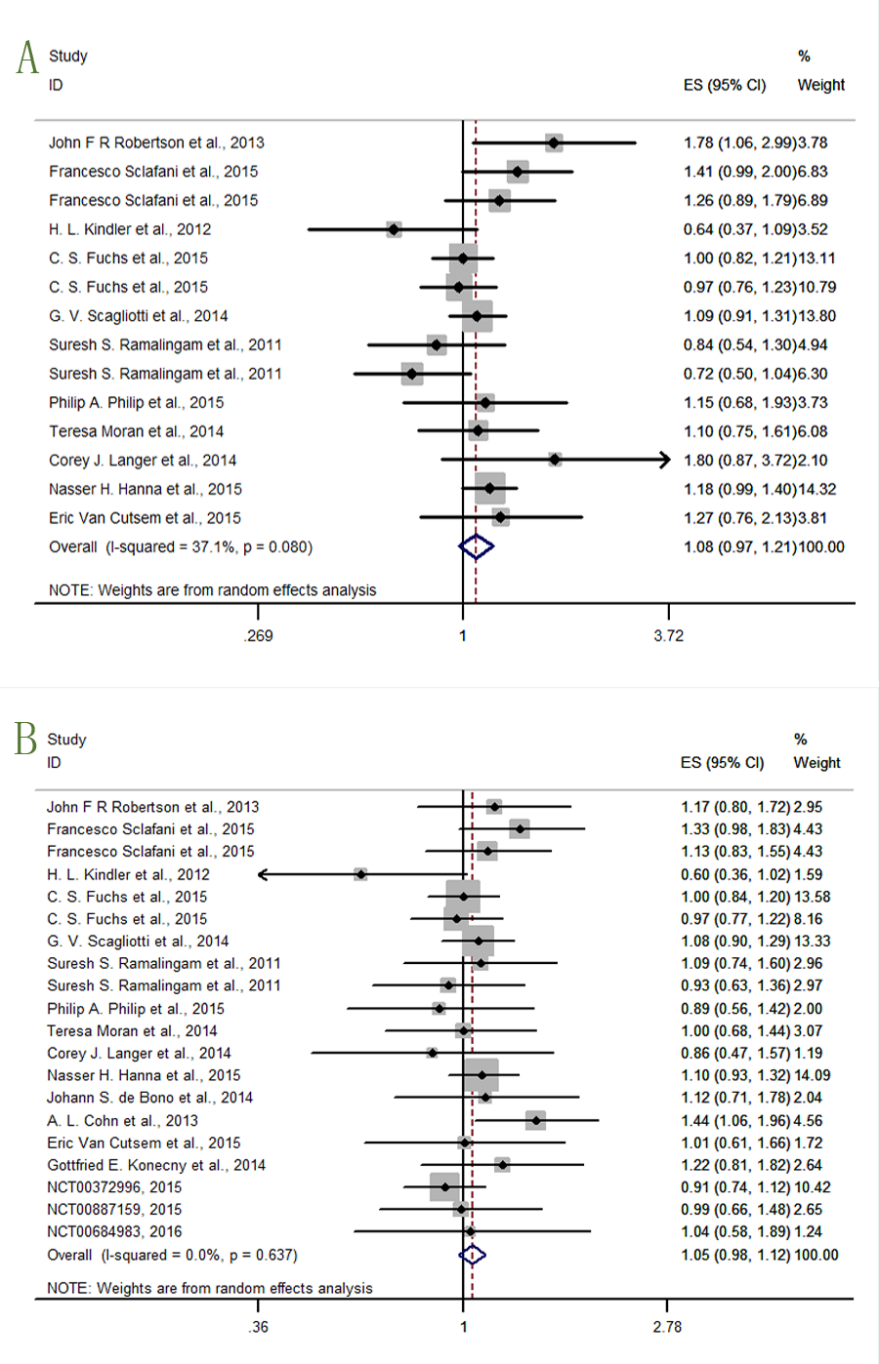
Forest plots of (A) hazard ratio (HR) for overall OS; (B) HRs for overall PFS

We got data of OS (overall survival) from eleven studies [12, 14, 16, 19–24], and PFS (progression-free survival) data from 17 studies [12–14, 16–22] (NCT00372996, 2015; NCT00887159, 2015; NCT00684983, 2016). The analysis results were shown in Figure 3 (OS: I^2^ = 37.1%, P = 0.080, HR = 1.08, 95% CI = 0.97-1.21; PFS: I^2^ = 0.0%, P = 0.637, HR = 1.05, 95% CI = 0.98-1.12), indicating that the relationship between prognosis and anti-IGF-1R agents was insignificant. But what's notable was that our analysis was not designed to draw definitive conclusions regarding efficacy, but rather to estimate the treatment effect on PFS and OS by IGF-1R anti-agents versus placebo.

In the OS analysis, the study Robertson JFR et al.,2013 [14] showed a significant result (HR = 1.78, 95% CI = 1.06-2.99) that the confidence interval (CI) range did not embrace 1. The result of lower confidence interval limit (lower CI limit)>1, and harzard ratio (HR)>1, indicated that the hazard risk rose by adding ganitumab (AMG-479). The rest studies lacked sufficient evidence to make conclusions, and that further analysis was required.

In the PFS analysis, only the study Cohn AL et al.,2013 [13] presented significant detrimental effect on cancer therapy (HR = 1.44, 95% CI = 1.06-1.96). The rest studies needed further analysis.

Based on the disappointing results, subgroup-analysis was conducted as follow.

### Subgroup analysis

In order to make out how insignificant results were forged, subgroup analysis was conducted in two aspects, the mAbs and the cancer types.

#### Subgroups allocated by anti-IGF-1R mAbs

**Figure 4:**
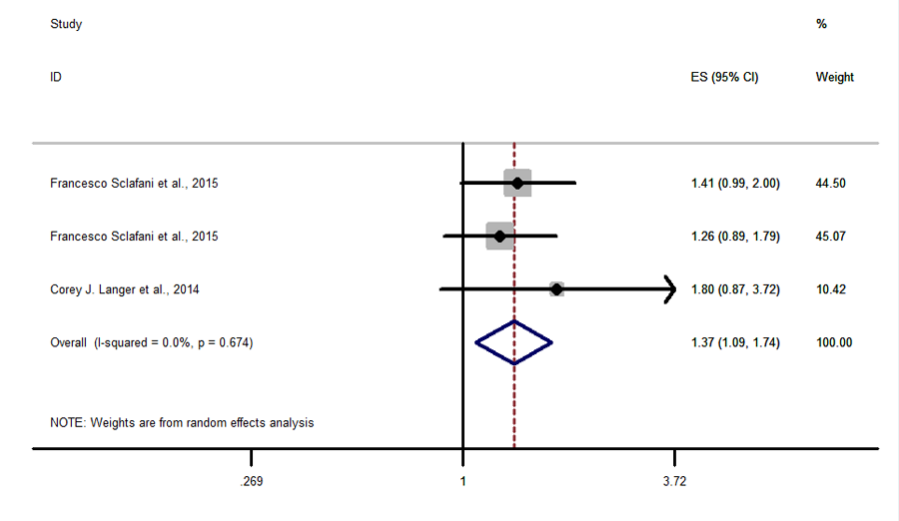
Forest plots of HRs with statistical significance for OS or PFS in the subgroups of patients allocated by Anti-IGF1R agents OS-dalotuzumab (MK-0646).

**Figure 5:**
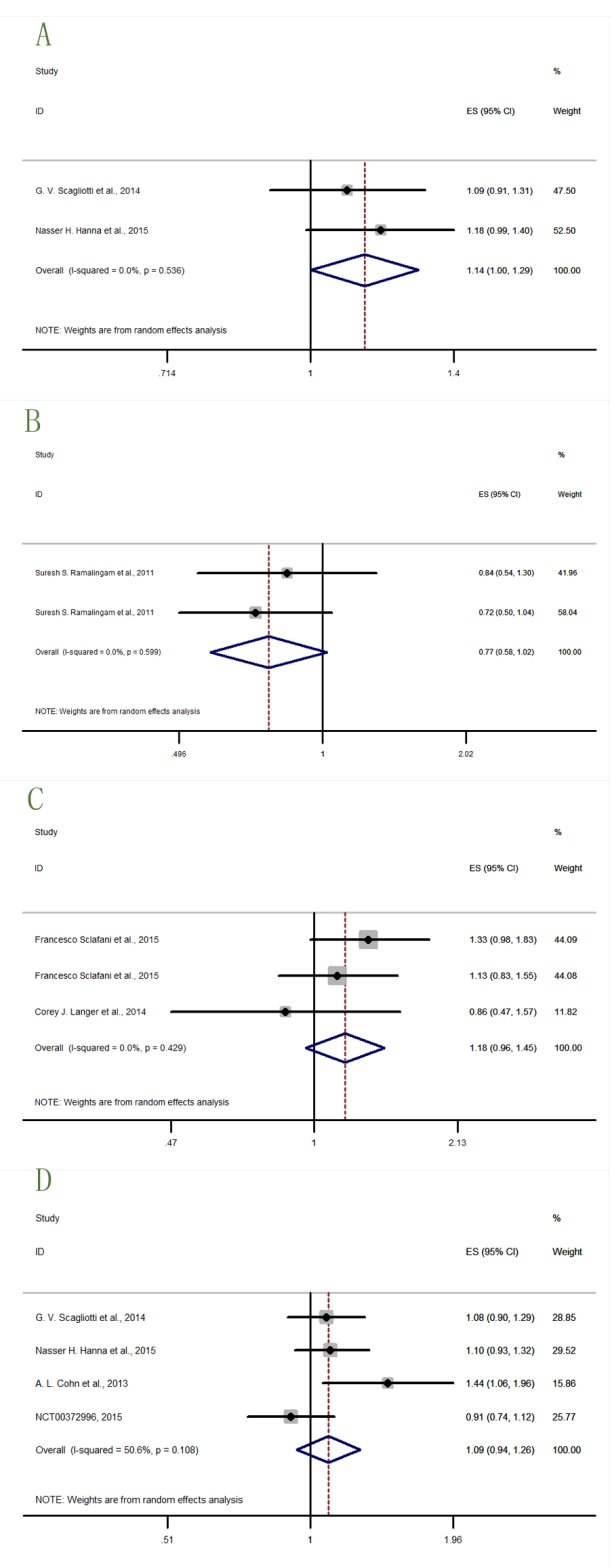
Forest plots of HRs with tendency but without statistical significance for OS or PFS in the subgroups of patients allocated by Anti-IGF1R agents **A**. OS-Figitumumab (CP-751,871); **B**. OS-Teprotumumab (R1507); **C**. PFS-dalotuzumab (MK-0646); **D**. PFS-Figitumumab (CP-751,871).

**Figure 6:**
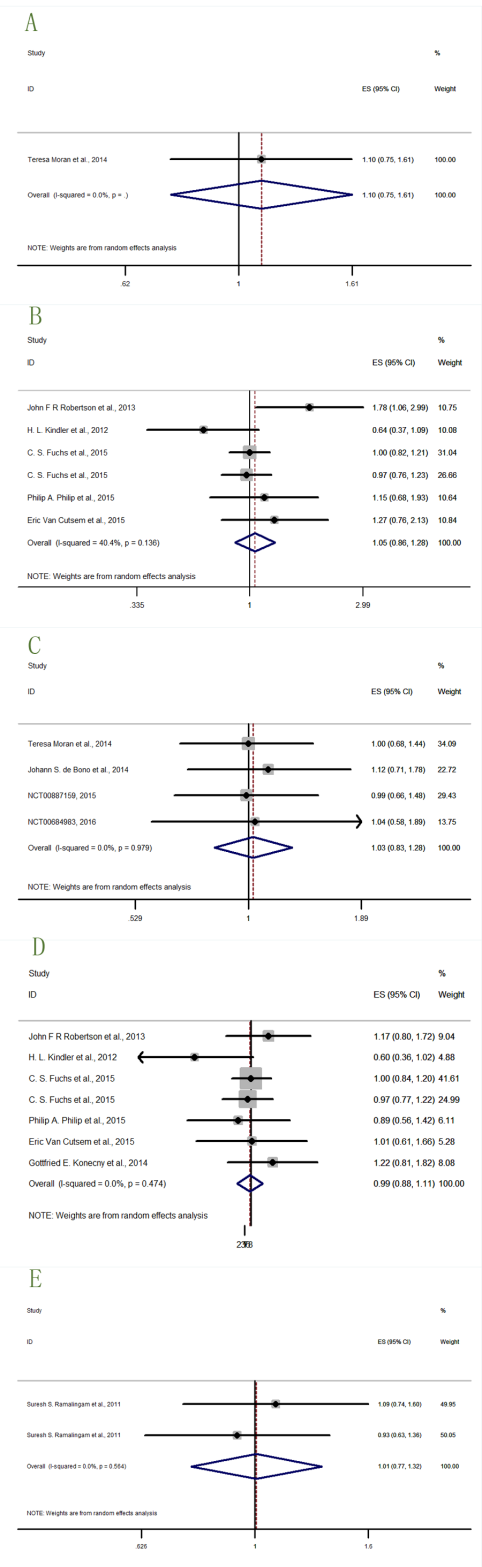
Forest plots of HRs with insignificance for OS or PFS in the subgroups of patients allocated by Anti-IGF1R agents **A**. OS-Cixutumumab (IMC-A12); **B**. OS-Ganitumab (AMG-479); **C**. PFS-Cixutumumab (IMC-A12); **D**. PFS-Ganitumab; **E**. PFS-Teprotumumab (R1507).

The study with significant result was shown in Figure 4, while studies with benefitial or detrimental trends but without statistical significance were shown in Figure 5, and studies with neither trending nor significant results were shown in Figure 6.

Three datas from two studies [15, 19] described the OS-dalotuzumab (MK-0646) in Figure 4, and the results (HR = 1.37, 95% CI = 1.09-1.74) indicated that dalotuzumab (MK-0646) should not be a suitable agents in cancer therapy.

Two study results [24, 25] (Figure 5A) described the OS-Figitumumab (CP-751,871) (HR = 1.14, 95% CI = 1.00-1.29), indicating that figitumumab (CP-751,871) may be harmful on cancer treatment. Two datas from one study [16] (Figure 5B) described the OS-Teprotumumab (R1507). The result (HR = 0.77, 95% CI = 0.55-1.02) hinted a trend that teprotumumab (R1507) may be beneficial to cancer therapy but not statistically significant. Certain conclusion needs further investigation to support. Three datas from two studies [15, 19] (Figure 5C) described the PFS-dalotuzumab (MK-0646), and four study datas [13, 24, 25] (NCT00372996, 2015) (Figure 5D) described the PFS-figitumumab (CP-751,871). The results showed worse trends but no statistical significance (HR = 1.18, 95% CI = 0.96-1.45; HR = 1.09, 95% CI = 0.94-1.26) for dalotuzumab (MK-0646) and figitumumab (CP-751,871) to treat cancer.

The OS-Cixutumumab (IMC-A12) was only insignificantly described by one study result (Teresa Moran et al., 2014) (Figure 6A) [20]. Six data from five studies [12, 14, 21–23] (Figure 6B) described the OS-ganitumab (AMG-479). The result (HR = 1.05, 95% CI = 0.85-1.28) was also insignificant. Four study datas [17, 20] (NCT00887159, 2015; NCT00684983, 2016) (Figure 6C) described the PFS-Cixutumumab (IMC-A12), seven datas from six studies [12, 14, 18, 21–23] (Figure 6D) described the PFS-ganitumab (AMG-479), and two datas from one study [16] (Figure 6E) described the PFS-teprotumumab (R1507). The results of these three subgroups (HR = 1.03, 95% CI = 0.83-1.28; HR = 0.99, 95% CI = 0.88-1.11; HR = 1.01, 95% CI = 0.77-1.32 respectively) were insignificant.

#### Allocated by cancer types

**Figure 7:**
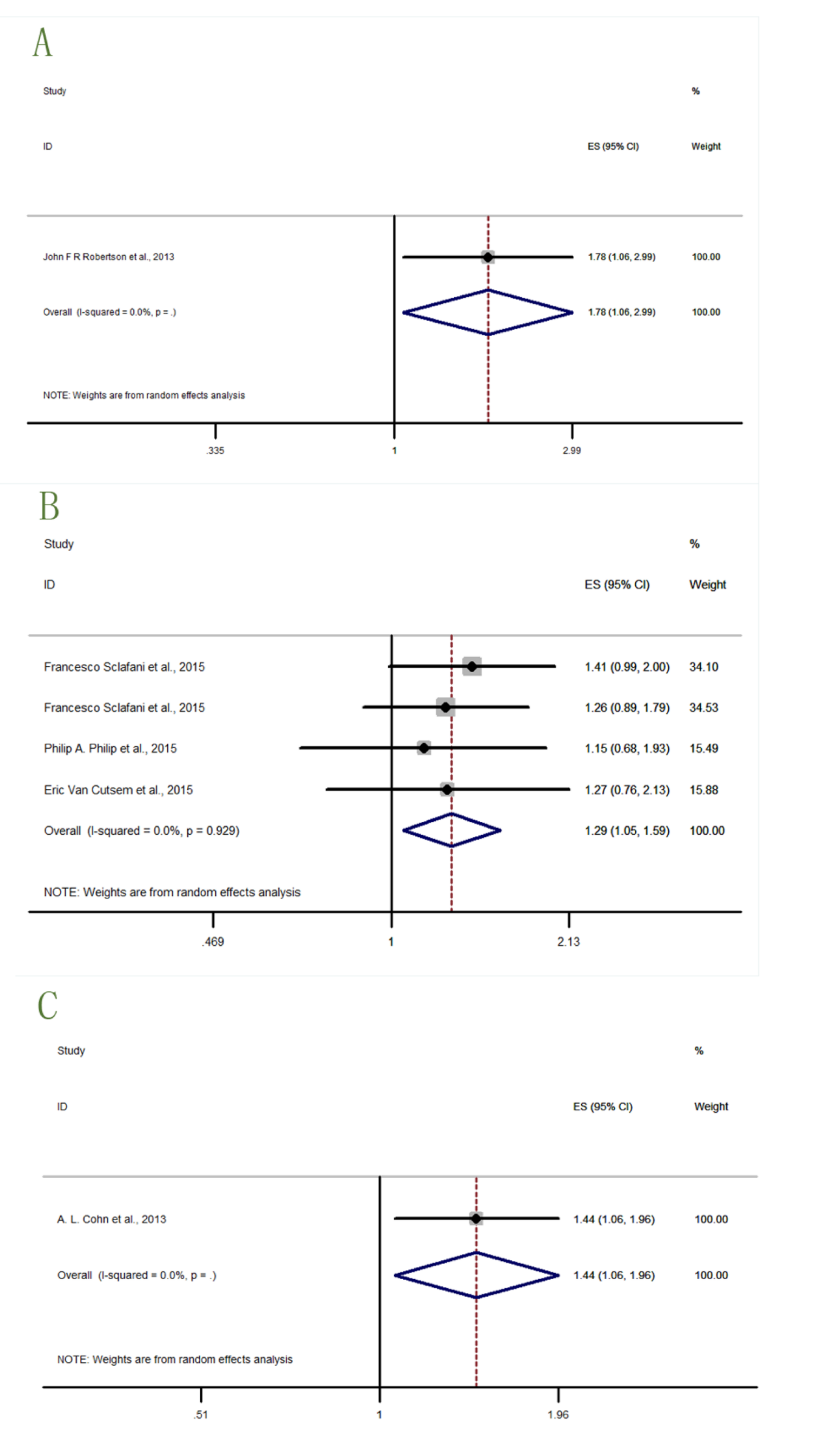
Forest plots of HRs with statistical significance for OS or PFS in the subgroups of patients allocated by cancer types **A**. OS-breast cancer; **B**. OS-colorectal cancer; **C**. PFS-prostate cancer.

**Figure 8:**
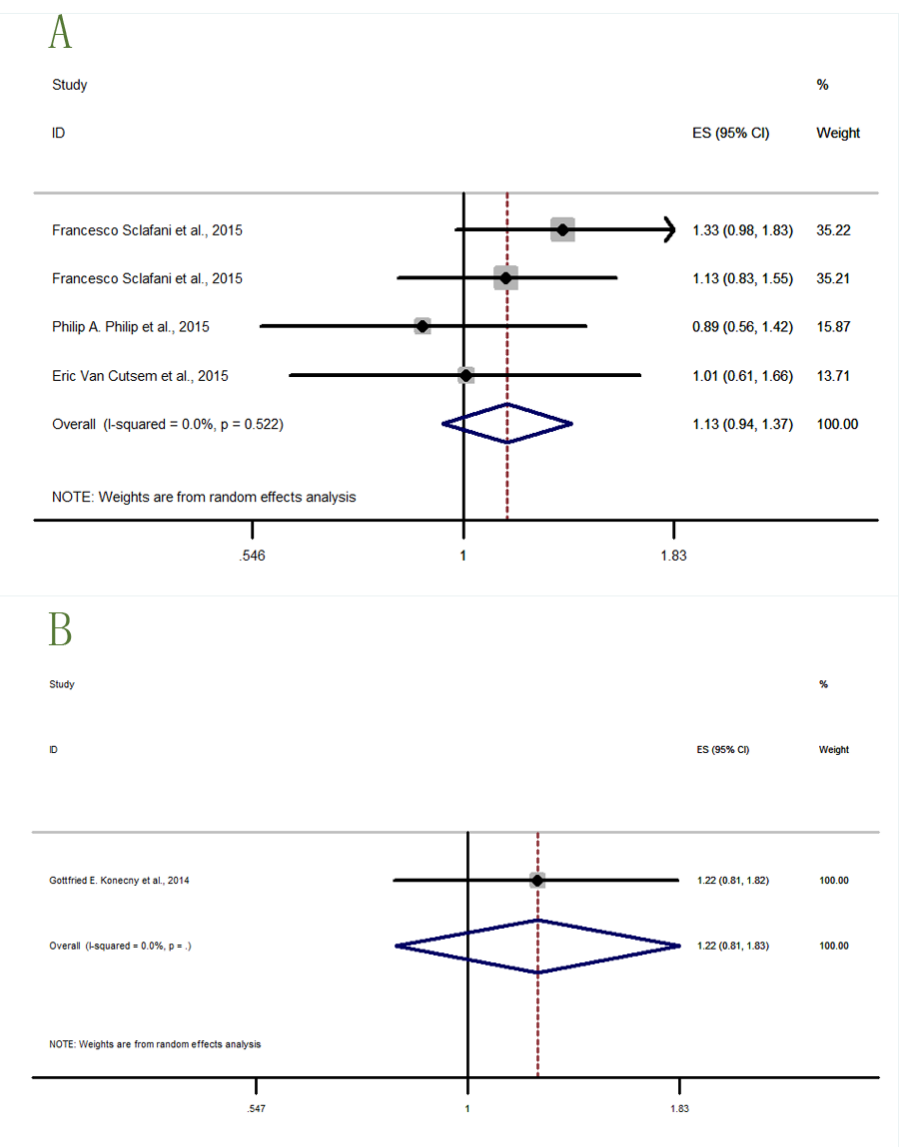
Forest plots of HRs with tendency but without statistical significance for OS or PFS in the subgroups of patients allocated by cancer types **A**. PFS-colorectal cancer; **B**. PFS-varian cancer.

**Figure 9:**
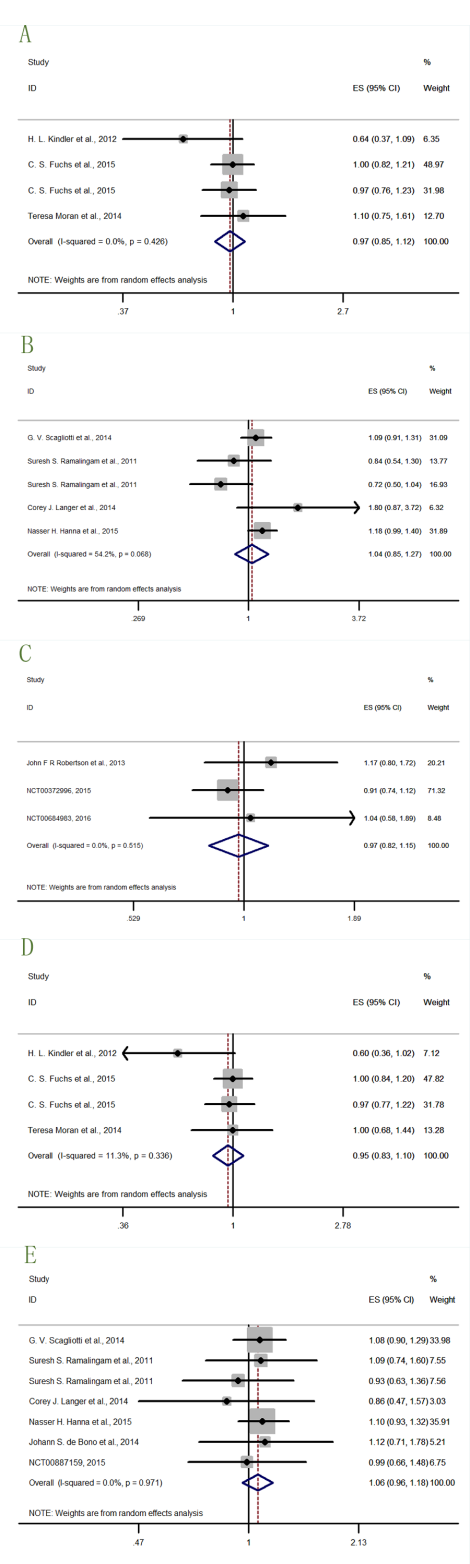
Forest plots of HRs with insignificance for OS or PFS in the subgroups of patients allocated by cancer types **A**. OS-pancreatic cancer; **B**. OS-lung cancer; **C**. PFS-breast cancer; **D**. PFS-pancreatic cancer; **E**. PFS-lung cancer.

Two cancer types (prostate cancer and ovarian cancer) lacked data to describe the OS. In Figure 7-8, the study with significant result was shown in Figure 7, while studies with detrimental trends but without statistical significance were shown in Figure 8, and studies with neither trending nor significant results were shown in Figure 9.

There was only one study [14] (Figure 7A) result (HR = 1.79, 95% CI = 1.08-2.99) describing the OS-breast cancer, indicating significantly detrimental effect for breast cancer treated with IGF-1R inhibitors. Study for breast cancer patients treated by anti-IGF-1R agents was unexpected. Four datas from three studies [15, 21, 22] (Figure 7B) described the OS-colorectal cancer (HR = 1.29, 95% CI = 1.05-1.59), revealing significantly worse effects on colorectal cancer patients that treated with IGF-1R inhibitors. Only one study result [13] (Figure 7C) described the PFS-prostate cancer (HR = 1.44, 95% CI = 1.06-1.98). The result significantly indicated the harmful effect of IGF-1R inhibitors to treat prostate cancer.

Four datas from three studies [15, 21, 22] (Figure 8A) described the PFS-colorectal cancer (HR = 1.13, 95% CI = 0.94-1.37), and only one study [18] (Figure 8B) described the PFS-ovarian cancer (HR = 1.22, 95% CI = 0.81-1.83). Two results did not have significant effects of IGF-1R inhibitors in cancer therapies but indicating a detrimental trends in survival outcomes.

Four datas from three studies [12, 20, 23] (Figure 9A) described the OS-pancreatic cancer (HR = 0.97, 95% CI = 0.85-1.12), and five datas from four studies [16, 19, 24, 25] (Figure 9B) described the OS-lung cancer (HR = 1.04, 95% CI = 0.85-1.27). These two results revealed insignificant curative effects of IGF-1R inhibitors in pancreatic and lung cancer treatment.

In the PFS results, there were three study results [14] (NCT00372996, 2015; NCT00684983, 2016) (Figure 9C) describing the PFS-breast cancer (HR = 0.97, 95% CI = 0.82-1.15), four datas from three studies [12, 20, 23] (Figure 9D) describing the PFS-pancreatic cancer (HR = 0.95, 95% CI = 0.83-1.10), and seven datas from six studies [16, 17, 19, 24, 25] (NCT00887159, 2015) (Figure 9E) describing the PFS-lung cancer (HR = 1.06, 95% CI = 0.96-1.18). These three results all insignificantly described the curative effects, thus further study of IGF-1R inhibitors to treat cancer was needed.

## DISCUSSION

Our analysis revealed the insignificant effects of IGF-1R inhibitors for solid tumor treatment so far. Harmful activity was even reported in some subgroups [13–15, 19, 21, 22], which was not accorded with the early study results [26, 27]. What's more, we noticed that in some trials the IGF-1R antibodies were well tolerated whereas in others they caused more severe side effects including hyperglycemia and neutropenia [14, 25].

It was meaningful to discuss the reasons of these phenomenon. As for the disappointing results, Sclafani F et al replied that it could be the potential of IGF-1R inhibition to accelerate tumour growth via aberrant feedback loops in intrinsically resistant tumours. This hypothesis would be supported in their study [15] by the higher dose intensity of dalotuzumab in arm A. Further more, they supposed it could be just a random effect. Robertson JFR et al considered that the limitations of study design may be of much concern, such as absence of an established biomarker and deficiency of hyperglycaemia as well as growth hormone concentrations measurement [14]. Another author Cohn AL et al thought that the disproportionately higher enrollment of patients with stage IV disease in the testing arm might have made the deviating outcomes. [13] Secondly, the adverse events could usually make the prognosis worse, but it was controversial. Robertson JFR et al found no safety issues that seemed to explain the negative efficacy findings [14], and the rate of serious adverse events reported in the study was similar to the rate reported in BOLERO-2 [28]. Moreover, a former work done by our laboratory also revealed the acceptability of adverse events caused by IGF-1R mAbs [29]. However, In the study conducted by de Bono JS et al [17], toxicity was substantially higher with testing group than with comparison group. And incidence of grade 3/4 treatment-related adverse events and severe adverse events (SAEs) increased, giving concern that the toxicity of combination treatment might detrimentally affect the results. There were other opinions to explain the pessimistic results. 1, Robertson JFR et al concluded in his study that ganitumab did not cross-react with insulin receptor and therefore did not inhibit IGF-2-mediated signaling via insulin receptor. IGF-1R inhibition alone was not sufficient if other pathways were activated (eg, EGFR) [14]. 2, Sclafani F et al analysed that poor recapitulation of tumor conditions as well as suboptimal patient selection might be the reasons [15]. 3, Wilson S et al thought It was possible that only in a minority of cases where IGF-1R was activated and exerted its oncogenic function [30], which turned targeting IGF-1R a subordinate method in some cases. 4, Garofalo C et al and Beltran PJ et al supposed a compensatory signaling existed via IGF2 through the insulin receptor [31, 32] which was not downregulated by ganitumab. 5, High frequency of downstream KRAS mutations in patients with pancreatic cancer was also considered as a potential explanation [21]. 6, Shin DH et al thought that targeting IR/IGF-1R was not enough to overcome growth and survival signals from downstream mutations in downstream pathways, such as PI3K/AKT axis. [33]

The diverse tolerabilities of IGF-1R mAbs in different trials refer to many possible mechanisms, most of which were unclear. We divided the enrolled studies into two groups by the median of any/severe adverse event rate. Then, we analyzed some potential factors to see the relationship between poor tolerabiliy group and good tolerability group [Supplement Table 1; Supplement Table 2]. The independent-sample t-test show that the adverse event rate are significantly different between poor tolerabiliy group and good tolerabiliy group in both Supplement Table 1 and Supplement Table 2. Then likelihood ratio chi-square test and independent-sample t-test are correspondingly used to analyze cancer types, mono-antibodies, regimens, patient number, and median age. The results indicate that the factors mentioned above are not associated with the diverse tolerability. Although in Supplement Table 2, some potential factors (e.g. cancer types and regimens) show statistically significant association with tolerability, we still hold the conservative estimates. Because the sample size are too small. More studies are suggested to confirm this problem. In all, we suggest that some more frequently observed severe adverse events for specific cancer types should be paid adequate attention when using mono-anti-IGF-1R mAbs, and it was essential to choose the proper combination regimen to reduce AE-occurrences.

### Suggestions on anti-IGF-1R mAb research and application

Most enrolled studies suggested researchers to select patients by specific biomarkers. In fact, there was an article providing evidence of the benefit to select patients by biomarkers [34]. Sclafani F et al found that high IGF-1 expression was predictive of poor outcome in the control arm but marked a subset of patients who appeared to benefit from the addition of weekly dalotuzumab to standard therapy. The study also showed that IGF-1 and IGF-2 might represent promising biomarkers predicting outcome with anti-IGF-1R- and anti-EGFR-targeted therapies [15]. Van Cutsem E et al investigated the correlation of efficacy endpoints with tumor MET, EGFR, PTEN, baseline circulating IGF1, IGF2, IGFBP1-3 and-6 protein levels. No strong evidence of predictive potential was found on efficacy endpoints for rilotumumab or ganitumab in combination with panitumumab [22]. The reasons might be the small number of patients per arm as well as potential imbalances in the arms for RAS mutations beyond KRAS exon 2. In study Cohn AL et al.,2013, PFS was associated with high circulating total IGF-1, IGF-2, and IGFBP-3, or associated with low IGFP-1 and IGFBP-2 in the ganitumab arm. In the placebo arm, low cytoplasmic PTEN expression was associated with longer PFS, indicating that the prognostic value of these markers requires further evaluation [13]. Based on the facts above, we recommend that anti-IGF-1R mAb studies should be conducted in biomarker-selected patients. Secondly, the combination regimen should consider the complementary efficacy of combination drugs on adverse effects. For example, hyperglycemia is the most common side effects of anti-IGF-1R mAbs [17, 35]. And metformin is an anti-diabetic drug with anti-cancer efficacy [36]. Thus, the combination of anti-IGF-1R mAbs and metformin may be a good choice.

### Conclusions

Our analysis used up-to-date data to show the pessimistic result of anti-IGF-1R mAbs on cancer therapy. However, it is too early to conclude IGF-IR antibodies have no utility as anti-cancer agents. It should be noted that up to November 2016, there were ongoing trials without data published (NCT01327612; NCT02306161; NCT01122199; NCT01061788; NCT01708161; NCT00791154; NCT01042379; NCT00769483; NCT01868997; NCT01232452; NCT00955305; NCT01142388). NCT01327612, NCT01122199, NCT01708161, NCT00769483, and NCT01868997 are in situation of “Active, not recruiting”. NCT01061788 and NCT01042379 are in situation of “Recruiting”. NCT00791154 and NCT01232452 are in situation of “completed” without any data published. While NCT02306161, NCT00955305, and NCT01142388 are in situation of “suspended”, “terminated with results”, and “Ongoing with results” respectively. NCT00955305 and NCT01142388 have posted the PFS and OS results in www.clinicaltrials.gov. However, the statistical analysis method of the two studies is Log Rank. And the PFS and OS results are also statistically insignificant. In NCT00955305, results for PFS and OS are p = 0.33 and p = 0.95 respectively, and in NCT01142388, the results are p = 0.58 and p = 0.50 respectively (all >0.05). Nevertheless, we still recommend the rest trials should be followed up in time, so that more precise conclusion that whether anti-IGF-1R-mAbs behave good as anti-cancer agents or not could be updated.

### Limitation

Firstly, The sources of enrolled studies were limited in Embase, Pubmed, Clinicaltrials.gov and other manual searching, which was not completely assured to cover all relevant data. Secondly, the data collected in our analysis were disappointing and the population were not large enough. Phase 3 clinical trials only occupied a small proportion in the enrolled studies. Moreover, Some enrolled studies possessed high risk of bias, which may, more or less, lead to deflection of the results.

## MATERIALS AND METHODS

### Publication search

We carried out a comprehensive systematic search of PubMed, EMBASE, and Clinicaltirals.gov (up to May 10, 2016). The following search key words were used to gain articles as comprehensive as possible: “ganitumab”, “AMG479”, ”dalotuzumab”, “MK 0646”, “cixutumumab”, “IMC-A12”, “Teprotumumab”, “R1507”, “figitumumab”, “CP751871”, “IGF-1R”, “Insulin-like growth factor-1 receptor”, “tumor”, “cancer”, “combination therapy”. Then subsequently, the searching strategy was used to identify the articles in relevance:”Search ( ( ( ( ( ( ( ( ( ( ( ( ( (Neoplasm [Title/Abstract]) OR Tumors [Title/Abstract]) OR Tumor [Title/Abstract]) OR Neoplasia [Title/Abstract]) OR Cancer [Title/Abstract]) OR Cancers [Title/Abstract]) OR Benign Neoplasms [Title/Abstract]) OR Neoplasms, Benign [Title/Abstract]) OR Benign Neoplasm [Title/Abstract]) OR Neoplasm, Benign [Title/Abstract]) OR Carcinoma [Title/Abstract]) OR Carcinomas [Title/Abstract])) AND ( ( ( ( ( ( ( ( ( ( ( ( ( ( ( ( ( ( ( ( ( ( ( ( ( ( ( ( ( ( ( ( ( ( ( ( ( ( ( ( ( ( (Combination Chemotherapy [Title/Abstract]) OR Drug Polytherapy [Title/Abstract]) OR Drug Polytherapies [Title/Abstract]) OR Polytherapies, Drug [Title/Abstract]) OR Polytherapy, Drug [Title/Abstract]) OR Therapy, Combination Drug [Title/Abstract]) OR Chemotherapy, Combination [Title/Abstract]) OR Chemotherapies, Combination [Title/Abstract]) OR Combination Chemotherapies [Title/Abstract]) OR Combination Drug Therapy [Title/Abstract]) OR Combination Drug Therapies [Title/Abstract]) OR Drug Therapies, Combination [Title/Abstract]) OR Therapies, Combination Drug [Title/Abstract]) OR Polychemotherapy [Title/Abstract]) OR Polychemotherapies [Title/Abstract]) OR Combined Antineoplastic Agents [Title/Abstract]) OR Antineoplastic Agents, Combined [Title/Abstract]) OR Agent, Combined Antineoplastic [Title/Abstract]) OR Agents, Combined Antineoplastic [Title/Abstract]) OR Antineoplastic Agent, Combined [Title/Abstract]) OR Combined Antineoplastic Agent [Title/Abstract]) OR Antineoplastic Combined Chemotherapy Regimens [Title/Abstract]) OR Drug Combinations, Antineoplastic [Title/Abstract]) OR Anticancer Drug Combinations [Title/Abstract]) OR Anticancer Drug Combination [Title/Abstract]) OR Drug Combination, Anticancer [Title/Abstract]) OR Drug Combinations, Anticancer [Title/Abstract]) OR Antineoplastic Drug Combinations [Title/Abstract]) OR Antineoplastic Drug Combination [Title/Abstract]) OR Combinations, Antineoplastic Drug [Title/Abstract]) OR Drug Combination, Antineoplastic [Title/Abstract]) OR Antineoplastic Chemotherapy Protocols [Title/Abstract]) OR Antineoplastic Chemotherapy Protocol [Title/Abstract]) OR Chemotherapy Protocol, Antineoplastic [Title/Abstract]) OR Protocol, Antineoplastic Chemotherapy [Title/Abstract]) OR Protocols, Antineoplastic Chemotherapy [Title/Abstract]) OR Cancer Chemotherapy Protocols [Title/Abstract]) OR Cancer Chemotherapy Protocol [Title/Abstract]) OR Chemotherapy Protocol, Cancer [Title/Abstract]) OR Chemotherapy Protocols, Cancer [Title/Abstract]) OR Protocol, Cancer Chemotherapy [Title/Abstract]) OR Protocols, Cancer Chemotherapy [Title/Abstract]) OR Chemotherapy Protocols, Antineoplastic [Title/Abstract])) AND ( ( ( ( ( ( ( ( ( ( ( ( ( ( ( ( ( ( ( (ganitumab [Title/Abstract]) OR AMG479 [Title/Abstract]) OR dalotuzumab [Title/Abstract]) OR MK 0646 [Title/Abstract]) OR MK0646 [Title/Abstract]) OR MK-0646 [Title/Abstract]) OR cixutumumab [Title/Abstract]) OR IMC-A12 [Title/Abstract]) OR Teprotumumab [Title/Abstract]) OR R 1507 [Title/Abstract]) OR R1507 [Title/Abstract]) OR R-1507 [Title/Abstract]) OR figitumumab [Title/Abstract]) OR CP751871 [Title/Abstract]) OR CP-751871 [Title/Abstract]) OR CP751,871 [Title/Abstract]) OR CP 751,871 [Title/Abstract]) OR CP-751,871 [Title/Abstract])) OR ( ( ( ( ( ( ( ( ( ( ( ( ( ( ( ( ( ( ( ( ( ( (IGF-1 Receptor) OR IGF 1 Receptor) OR Receptor, IGF-1) OR IGF-I Receptor) OR IGF I Receptor) OR Receptors, Insulin-Like-Growth Factor I) OR Receptor, IGF-I [Title/Abstract]) OR Receptor, IGF I [Title/Abstract]) OR Receptor, Insulin-Like Growth Factor I [Title/Abstract]) OR Receptor, Insulin-Like Growth Factor Type 1 [Title/Abstract]) OR Receptors, IGF-1 [Title/Abstract]) OR IGF-1 Receptors [Title/Abstract]) OR Receptors, IGF 1 [Title/Abstract]) OR IGF Type 1 Receptor [Title/Abstract]) OR Insulin-Like-Growth Factor I Receptor [Title/Abstract]) OR Insulin Like Growth Factor I Receptor [Title/Abstract]) OR Receptor, IGF Type 1 beta Subunit [Title/Abstract]) OR Receptor, IGF Type 1 alpha Subunit [Title/Abstract]) OR Insulin-Like Growth Factor Receptors [Title/Abstract]) OR Insulin Like Growth Factor Receptors [Title/Abstract]) OR Insulin-Like Growth Factor Receptor [Title/Abstract]) OR Receptors, Insulin-Like Growth Factors [Title/Abstract]) OR Receptors, Insulin Like Growth Factors [Title/Abstract]))”. There are no restrictions on the types of studies and only publications published by English were included. The bibliographies of eligible studies were searched by hand for other relevant articles. The studies were selected following the steps in sequence: 1), Browse the tittles and eliminate irrelevant articles; 2), Skim the abstracts of the rest articles, and pick out those who satisfied the exclusion criteria and keep the ones accord with inclusion criteria; 3), Finally read the left articles and extract the data and information.

### Inclusion criteria

The inclusion criteria to obtain eligible studies: i), Studies that evaluated the efficacy of IGF-1R inhibitors by OS, PFS or both of them were eligible to be included. ii), Studies who contained two or more than two pallelel arms were included. Moreover, IGF-1R inhibitors must exert as controlled factors. iii), Studies that could be found with full articles or without full articles published but useful data was sufficient in Clinicaltrials.gov were included. iv), When the results were obtained from the same population and were published in several publications, only the most recent report or most informative one was included.

### Exclusion criteria

The exclusion criteria to exclude the ineligible studies: i), Studies without OS and PFS data. ii), The allocation method did not reveal the contral relationship but only escalation relationship among cohorts. iii), Studies of single group clinical assignment were excluded. iv), Those who were not cllinical studies were excluded.

### Assessment of risk of bias

The risk of bias assessment was important for the quality of analysis. Therefore, we assessed risk of bias by Cochrane Collaboration's tool from six key bias domains: selection bias, performance bias, detection bias, attrition bias, reporting bias and other bias. [37] The authors’ judgements for a trial included low, unclear and high risk. They were defined as followed: Low risk of bias refers to the bias that is unlikely to alter the results seriously; Unclear risk of bias raises some doubt about the results; And the high risk of bias may alter the results seriously. Within a trial, judgement of low risk refered to low risk of bias for all key domains; Unclear risk of bias satisfied with low or unclear risk of bias for all key domains. And high risk of bias refered to high risk of bias for one or more key domains. The assessment result was shown in Table 1. The study John F R Robertson et al., 2013 was assessed the best quality. The rest studies were assessed as unclear with exception of two high-risk studies (Nasser H. Hanna et al., 2015; H. L. Kindler et al., 2012).

All studies included in our analysis were assessed by two reviewers. When it came to discrepancies, the two reviewers decided to include or exclude studies after joint review.

### Statistical analysis

The primary endpoint was PFS and the secondary endpoints was OS. The Progression-free survival (PFS) is a measure of treatment efficacy on a disease. It is the time that passes from a certain date (generally the first day of treatment, or the day in which a patient is enrolled in a clinical trial) to the date on which disease “progresses” or the date on which the patient dies, from any cause. The OS (also called overall survival) is the length of time from either the date of diagnosis or the start of treatment for a disease, such as cancer. In our analysis, measuring the PFS and OS is one way to see how well the IGF-1R inhibitors work for the patients suffering from solid tumors. In order to find an appropriate calculation model, We conducted heterogeneity analysis. Heterogeneity assumption was assessed by the I^2^ statistic and directed the analysis to be conducted in a random-effects model. Sensitivity analyses were conducted by removing one study each time. [38] Potential publication bias was evaluated by Begg's funnel plots and if the funnel plot showed asymmetry, it suggested a possible publication bias. And p≤0.05 used to assess the heterogeneity suggested statistically significantly bias in two-tailed level. All the statistical tests were performed with Stata 12.0 software.

## SUPPLEMENTARY MATERIALS AND TABLES



## References

[1] Siegel RL, Miller KD, Jemal A (2015). Cancer statistics, 2015. CA: A Cancer Journal for Clinicians.

[2] Kurmasheva RT, Houghton PJ (2006). IGF-I mediated survival pathways in normal and malignant cells. Biochim Biophys Acta.

[3] Samani AA, Yakar S, LeRoith D, Brodt P (2007). The Role of the IGF System in Cancer Growth and Metastasis: Overview and Recent Insights. Endocrine Reviews.

[4] Heidegger I, Pircher A, Klocker H, Massoner P (2011). Targeting the insulin-like growth factor network in cancer therapy. Cancer Biol Ther.

[5] Hewish M, Chau I, Cunningham D (2009). Insulin-like growth factor 1 receptor targeted therapeutics: novel compounds and novel treatment strategies for cancer medicine. Recent Pat Anticancer Drug Discov.

[6] King ER, Wong KK (2012). Insulin-like growth factor: current concepts and new developments in cancer therapy. Recent Pat Anticancer Drug Discov.

[7] Arcaro A (2013). Targeting the insulin-like growth factor-1 receptor in human cancer. Front Pharmacol.

[8] Navarro M, Baserga R (2001). Limited redundancy of survival signals from the type 1 insulin-like growth factor receptor. Endocrinology.

[9] Park S, Chapuis N, Tamburini J, Bardet V, Cornillet-Lefebvre P, Willems L, Green A, Mayeux P, Lacombe C, Bouscary D (2010). Role of the PI3K/AKT and mTOR signaling pathways in acute myeloid leukemia. Haematologica.

[10] Osaki M, Oshimura M, Ito H (2004). PI3K-Akt pathway: its functions and alterations in human cancer. Apoptosis.

[11] Hadari YR, Tzahar E, Nadiv O, Rothenberg P, Roberts CT, LeRoith D, Yarden Y, Zick Y (1992). Insulin and insulinomimetic agents induce activation of phosphatidylinositol 3′-kinase upon its association with pp185 (IRS-1) in intact rat livers. J Biol Chem.

[12] Kindler HL, Richards DA, Garbo LE, Garon EB, Stephenson JJ, Rocha-Lima CM, Safran H, Chan D, Kocs DM, Galimi F, McGreivy J, Bray SL, Hei Y (2012). A randomized, placebo-controlled phase 2 study of ganitumab (AMG 479) or conatumumab (AMG 655) in combination with gemcitabine in patients with metastatic pancreatic cancer. Ann Oncol.

[13] Cohn AL, Tabernero J, Maurel J, Nowara E, Sastre J, Chuah BY, Kopp MV, Sakaeva DD, Mitchell EP, Dubey S, Suzuki S, Hei YJ, Galimi F (2013). A randomized, placebo-controlled phase 2 study of ganitumab or conatumumab in combination with FOLFIRI for second-line treatment of mutant KRAS metastatic colorectal cancer. Ann Oncol.

[14] Robertson JF, Ferrero JM, Bourgeois H, Kennecke H, de Boer RH, Jacot W, McGreivy J, Suzuki S, Zhu M, McCaffery I, Loh E, Gansert JL, Kaufman PA (2013). Ganitumab with either exemestane or fulvestrant for postmenopausal women with advanced, hormone-receptor-positive breast cancer: a randomised, controlled, double-blind, phase 2 trial. Lancet Oncol.

[15] Sclafani F, Kim TY, Cunningham D, Kim TW, Tabernero J, Schmoll HJ, Roh JK, Kim SY, Park YS, Guren TK, Hawkes E, Clarke SJ, Ferry D (2015). A Randomized Phase II/III Study of Dalotuzumab in Combination With Cetuximab and Irinotecan in Chemorefractory, KRAS Wild-Type, Metastatic Colorectal Cancer. J Natl Cancer Inst.

[16] Ramalingam SS, Spigel DR, Chen D, Steins MB, Engelman JA, Schneider CP, Novello S, Eberhardt WE, Crino L, Habben K, Liu L, Janne PA, Brownstein CM (2011). Randomized phase II study of erlotinib in combination with placebo or R1507, a monoclonal antibody to insulin-like growth factor-1 receptor, for advanced-stage non-small-cell lung cancer. J Clin Oncol.

[17] de Bono JS, Piulats JM, Pandha HS, Petrylak DP, Saad F, Aparicio LM, Sandhu SK, Fong P, Gillessen S, Hudes GR, Wang T, Scranton J, Pollak MN (2014). Phase II randomized study of figitumumab plus docetaxel and docetaxel alone with crossover for metastatic castration-resistant prostate cancer. Clin Cancer Res.

[18] Konecny GE, Haluska P, Janicke F, Sehouli J, Beckmann MW, Feisel G, Polcher M, Roman L, Rody A, Karlan B, Ray-Coquard IL, Provencher DM, Ben-Baruch N (2014). A phase II, multicenter, randomized, double-blind, placebo-controlled trial of ganitumab or placebo in combination with carboplatin/paclitaxel as front-line therapy for optimally debulked primary ovarian cancer: The TRIO14 trial. J Clin Oncol (Meeting Abstracts).

[19] Langer CJ, Novello S, Park K, Krzakowski M, Karp DD, Mok T, Benner RJ, Scranton JR, Olszanski AJ, Jassem J (2014). Randomized, phase III trial of first-line figitumumab in combination with paclitaxel and carboplatin versus paclitaxel and carboplatin alone in patients with advanced non-small-cell lung cancer. J Clin Oncol.

[20] Moran T, Felip E, Keedy V, Borghaei H, Shepherd FA, Insa A, Brown H, Fitzgerald T, Sathyanarayanan S, Reilly JF, Mauro D, Hsu K, Yan L (2014). Activity of dalotuzumab, a selective anti-IGF1R antibody, in combination with erlotinib in unselected patients with Non-small-cell lung cancer: a phase I/II randomized trial. Exp Hematol Oncol.

[21] Philip PA, Goldman B, Ramanathan RK, Lenz HJ, Lowy AM, Whitehead RP, Wakatsuki T, Iqbal S, Gaur R, Benedetti JK, Blanke CD (2014). Dual blockade of epidermal growth factor receptor and insulin-like growth factor receptor-1 signaling in metastatic pancreatic cancer: phase Ib and randomized phase II trial of gemcitabine, erlotinib, and cixutumumab versus gemcitabine plus erlotinib (SWOG S0727). Cancer.

[22] Van Cutsem E, Eng C, Nowara E, Swieboda-Sadlej A, Tebbutt NC, Mitchell E, Davidenko I, Stephenson J, Elez E, Prenen H, Deng H, Tang R, McCaffery I (2014). Randomized phase Ib/II trial of rilotumumab or ganitumab with panitumumab versus panitumumab alone in patients with wild-type KRAS metastatic colorectal cancer. Clin Cancer Res.

[23] Fuchs CS, Azevedo S, Okusaka T, Van Laethem JL, Lipton LR, Riess H, Szczylik C, Moore MJ, Peeters M, Bodoky G, Ikeda M, Melichar B, Nemecek R (2015). A phase 3 randomized, double-blind, placebo-controlled trial of ganitumab or placebo in combination with gemcitabine as first-line therapy for metastatic adenocarcinoma of the pancreas: the GAMMA trial. Ann Oncol.

[24] Hanna NH, Dahlberg SE, Kolesar JM, Aggarwal C, Hirsch FR, Ramalingam SS, Schiller JH (2015). Three-arm, randomized, phase 2 study of carboplatin and paclitaxel in combination with cetuximab, cixutumumab, or both for advanced non-small cell lung cancer (NSCLC) patients who will not receive bevacizumab-based therapy: An Eastern Cooperative Oncology Group (ECOG) study (E4508). Cancer.

[25] Scagliotti GV, Bondarenko I, Blackhall F, Barlesi F, Hsia TC, Jassem J, Milanowski J, Popat S, Sanchez-Torres JM, Novello S, Benner RJ, Green S, Molpus K (2015). Randomized, phase III trial of figitumumab in combination with erlotinib versus erlotinib alone in patients with nonadenocarcinoma nonsmall-cell lung cancer. Ann Oncol.

[26] Schoffski P, Adkins D, Blay JY, Gil T, Elias AD, Rutkowski P, Pennock GK, Youssoufian H, Gelderblom H, Willey R, Grebennik DO (2013). An open-label, phase 2 study evaluating the efficacy and safety of the anti-IGF-1R antibody cixutumumab in patients with previously treated advanced or metastatic soft-tissue sarcoma or Ewing family of tumours. Eur J Cancer.

[27] Rajan A, Carter CA, Berman A, Cao L, Kelly RJ, Thomas A, Khozin S, Chavez AL, Bergagnini I, Scepura B, Szabo E, Lee MJ, Trepel JB (2014). Cixutumumab for patients with recurrent or refractory advanced thymic epithelial tumours: a multicentre, open-label, phase 2 trial. Lancet Oncol.

[28] Baselga J, Campone M, Piccart M, Burris HA, Rugo HS, Sahmoud T, Noguchi S, Gnant M, Pritchard KI, Lebrun F, Beck JT, Ito Y, Yardley D (2012). Everolimus in postmenopausal hormone-receptor-positive advanced breast cancer. N Engl J Med.

[29] Ma H, Zhang T, Shen H, Cao H, Du J (2014). The adverse events profile of anti-IGF-1R monoclonal antibodies in cancer therapy. Br J Clin Pharmacol.

[30] Wilson S, Chia SK (2013). IGF-1R inhibition: right direction, wrong pathway?. Lancet Oncol.

[31] Beltran PJ, Chung YA, Moody G, Mitchell P, Cajulis E, Vonderfecht S, Kendall R, Radinsky R, Calzone FJ (2011). Efficacy of ganitumab (AMG 479), alone and in combination with rapamycin, in Ewing’s and osteogenic sarcoma models. J Pharmacol Exp Ther.

[32] Garofalo C, Manara MC, Nicoletti G, Marino MT, Lollini PL, Astolfi A, Pandini G, Lopez-Guerrero JA, Schaefer KL, Belfiore A, Picci P, Scotlandi K (2011). Efficacy of and resistance to anti-IGF-1R therapies in Ewing’s sarcoma is dependent on insulin receptor signaling. Oncogene.

[33] Shin DH, Min HY, El-Naggar AK, Lippman SM, Glisson B, Lee HY (2011). Akt/mTOR counteract the antitumor activities of cixutumumab, an anti-insulin-like growth factor I receptor monoclonal antibody. Mol Cancer Ther.

[34] Palmisano WA, Divine KK, Saccomanno G, Gilliland FD, Baylin SB, Herman JG, Belinsky SA (2000). Predicting lung cancer by detecting aberrant promoter methylation in sputum. Cancer Res.

[35] Becerra CR, Salazar R, Garcia-Carbonero R, Thomas AL, Vazquez-Mazon FJ, Cassidy J, Maughan T, Castillo MG, Iveson T, Yin D, Green S, Bergsland EK (2014). Figitumumab in patients with refractory metastatic colorectal cancer previously treated with standard therapies: a nonrandomized, open-label, phase II trial. Cancer Chemother Pharmacol.

[36] Martin M, Marais R (2012). Metformin: a diabetes drug for cancer, or a cancer drug for diabetics?. J Clin Oncol.

[37] Higgins JP, Altman DG, Gotzsche PC, Juni P, Moher D, Oxman AD, Savovic J, Schulz KF, Weeks L, Sterne JA (2011). The Cochrane Collaboration’s tool for assessing risk of bias in randomised trials. Bmj.

[38] Yang ZY, Wu XY, Huang YF, Di MY Zheng DY, Chen JZ, Ding H, Mao C, Tang JL (2013). Promising biomarkers for predicting the outcomes of patients with KRAS wild-type metastatic colorectal cancer treated with anti-epidermal growth factor receptor monoclonal antibodies: a systematic review with meta-analysis. Int J Cancer.

