# Supplementary material for: Update of IGF-1 receptor inhibitor (ganitumab, dalotuzumab, cixutumumab, teprotumumab and figitumumab) effects on cancer therapy
Source: Oncotarget. 2017 Feb 25;8(17):29501–18. doi: 10.18632/oncotarget.15704 (PMC5438747; doi:10.18632/oncotarget.15704)
Supplement: Supplementary file 1 [file oncotarget-08-29501-s001.pdf]

Update of IGF-1 receptor inhibitor (Ganitumab, Dalotuzumab, Cixutumumab, Teprotumumab and Figitumumab) effects on cancer therapy

Supplementary Material

Supplement Table 1: Potential factors associated with any adverse events rate.

groups	Poor tolerability group								Good tolerability group									Stat method	P
Study ID	1	6	15	11	5	17	18	19	14	10	20	13	7	16	4	3	2		
Any AES rate	0.99	0.99	0.99	0.98	0.98	0.98	0.98	0.98	0.96	0.96	0.96	0.94	0.93	0.92	0.85	0.77	0.68	t test*	<0.05
Cancer types	Bre	Pan	Pro	Pan	Pan	Ova	Bre	Lun	Lun	Col	Bre	Lun	Lun	Col	Pan	Col	Col	Chi-Square#	0.058
Mono-mAbs	Gan	Gan	Fig	Cix	Gan	Gan	Fig	Cix	Cix	Gan	Cix	Fig	Fig	Gan	Gan	Dal	Dal	Chi-Square#	0.414
Regimen ID	1	2	3	4	2	5	15	14	6	7	16	8	9	10	2	11	11	Chi-Square#	0.144
Patients NO.	106	160	97	57	315	88	115	52	48	46	45	338	289	51	40	119	119	t test*	0.312
Median age	61	62	68.9	63	62	58	61.2	64	60	62	53	62	62	58	66	LA	LA	t test*	0.093

Supplement Table 2: Potential factors associated with Severe adverse events rate.

groups	Poor tolerability group										Good tolerability group										Stat method	P
Study ID	19	13	14	5	15	7	11	6	4	16	2	3	1	12	17	18	8	20	9	10		
Severe AES rate	0.9	0.83	0.75	0.68	0.66	0.63	0.6	0.59	0.58	0.55	0.5	0.42	0.39	0.35	0.34	0.33	0.32	0.31	0.25	0.2	t test*	<0.05
Cancer types	Lun	Lun	Lun	Pan	Pro	Lun	Pan	Pan	Pan	Col	Col	Col	Bre	Lun	Ova	Bre	Lun	Bre	Lun	Col	Chi-Square#	<0.05
Mono-mAbs	Cix	Fig	Cix	Gan	Fig	Fig	Cix	Gan	Gan	Gan	Dal	Dal	Gan	Dal	Gan	Fig	Tep	Cix	Tep	Gan	Chi-Square#	0.057
Regimen ID	14	8	6	2	3	9	4	2	2	10	11	11	1	12	5	15	13	16	13	7	Chi-Square#	<0.05
Patients NO.	52	338	48	315	97	289	57	160	40	51	119	119	106	37	88	115	57	45	57	46	t test*	0.608
Median age	64	62	60	62	68.9	62	63	62	66	58	LA	LA	61	62	58	61.2	63	53	62	62	t test*	0.826
